# Statistical inference for diagnostic test accuracy studies with multiple comparisons

**DOI:** 10.1177/09622802241236933

**Published:** 2024-03-15

**Authors:** Max Westphal, Antonia Zapf

**Affiliations:** 1 201971 Fraunhofer Institute for Digital Medicine MEVIS, Bremen, Germany; 2 Department of Medical Biometry and Epidemiology, 37734 University Medical Center Hamburg-Eppendorf, Hamburg, Germany; * The two authors contributed equally and are listed in alphabetical order

**Keywords:** Diagnosis, medical testing, multiple testing, model selection, prediction, prognosis

## Abstract

Diagnostic accuracy studies assess the sensitivity and specificity of a new index test in relation to an established comparator or the reference standard. The development and selection of the index test are usually assumed to be conducted prior to the accuracy study. In practice, this is often violated, for instance, if the choice of the (apparently) best biomarker, model or cutpoint is based on the same data that is used later for validation purposes. In this work, we investigate several multiple comparison procedures which provide family-wise error rate control for the emerging multiple testing problem. Due to the nature of the co-primary hypothesis problem, conventional approaches for multiplicity adjustment are too conservative for the specific problem and thus need to be adapted. In an extensive simulation study, five multiple comparison procedures are compared with regard to statistical error rates in least-favourable and realistic scenarios. This covers parametric and non-parametric methods and one Bayesian approach. All methods have been implemented in the new open-source R package cases which allows us to reproduce all simulation results. Based on our numerical results, we conclude that the parametric approaches (maxT and Bonferroni) are easy to apply but can have inflated type I error rates for small sample sizes. The two investigated Bootstrap procedures, in particular the so-called pairs Bootstrap, allow for a family-wise error rate control in finite samples and in addition have a competitive statistical power.

## 1. Introduction

The aim of diagnostic and prognostic studies is generally to differentiate between two groups, for example, the diseased from the non-diseased or those with good outcome from those with poor outcome. This differentiation can be based on a single clinical parameter or a combination of parameters. The methods and results presented here are independent of whether the goal is diagnosis or prognosis and whether a single parameter or a combination is evaluated. Furthermore, this work also covers the scenario that several investigated diagnostic procedures are defined by different (machine-learned) prediction models which are based on the same input data, for example, medical images. For better readability, the terms diagnosis, diagnostic study and diagnostic test are used in the following, but prognosis, prognosis study and prognostic marker are meant at the same time.

In order to assess the accuracy of a new diagnostic test, the so-called index test, the result is compared with the true condition, which is assessed using the gold standard or reference standard. The recommended co-primary endpoints are sensitivity as the proportion correctly diagnosed as diseased and specificity as the proportion correctly diagnosed as non-diseased. If a standard test exists, the index test should be compared with it, preferably in the within-subject design (all tests in all individuals). However, if there is no established comparator, the aim is to demonstrate a pre-specified minimum sensitivity and specificity, the so-called minimally acceptable criteria.^1,2^

Since the study is only considered a success if both hypotheses regarding sensitivity and specificity can be rejected, no correction is required for the multiplicity of the two endpoints.^1^ However, multiplicity problems may occur elsewhere in diagnostic studies, especially if the aim is to evaluate different markers in parallel and select the best diagnostic test or to determine the optimal cut-off value for a single marker. Usually, a test or cut-off value is selected in a first step data-driven and then validated in a new study. However, it is known that data-driven selection may lead to an overestimation of diagnostic accuracy and that the results of the selection study often cannot be replicated in the validation study.^3^ The same phenomenon, sometimes referred to as optimization bias or selection-induced bias, is well known to also exist in bioinformatics and predictive modelling.^4,5^

A framework for the evaluation of multiple prediction models that address this multiplicity issue has been proposed recently.^6,7^ Hereby, not only the (apparently) single-best model is chosen and subsequently validated, but rather a set of promising models from which the best is selected in the evaluation study. An adaptation of this approach to diagnostic accuracy studies with co-primary endpoints sensitivity and specificity is possible.^8^ In the same way, the framework can also be used to determine the optimal cut-off value for a single biomarker after choosing a range of promising cut points in a prior development study. Regardless of the context, this framework offers increased flexibility and can increase statistical power, that is, the probability of correctly demonstrating a sufficiently high diagnostic accuracy for one of the selected tests.

However, it is important to correct for multiplicity in the validation study to avoid inflation of the type-one error. The correction approaches can be divided into single-step and stepwise procedures. In contrast to stepwise procedures, single-step procedures have the advantage that associated simultaneous confidence intervals can be constructed.^9^ This is relevant in diagnostic accuracy studies as confidence intervals are an important indicator of estimation uncertainty.

The simplest single-step correction is the Bonferroni adjustment, in which the adjusted significance level is equal to the global significance level, say α=0.05 , divided by the number of tests. This approach leads to conservative results when the diagnostic test results are positively correlated. This is for instance the case if the same biomarker or risk score is dichotomized at slightly different cut points. More elaborate multiple comparison procedures considered in this work aim to exploit the correlation structure and thereby increase statistical power. Existing approaches include the maxT-approach,^10^ which has already been utilized.^6,7^ This method is based on a multivariate normal approximation which leads to less conservative decisions in light of positive correlations. Nonparametric methods^11^ for diagnostic test evaluation have been transferred to early diagnostic studies with the area under the receiver operating characteristic (ROC) curve (AUC) as a primary outcome measure.^12^ This approach was also adapted to studies with sensitivity and specificity as co-primary endpoints.^13^

The main contribution of this work is the adaptation of several multiple comparison procedures to diagnostic accuracy studies with multiple index tests with sensitivity and specificity as co-primary endpoints. The results of an extensive simulation study are presented to allow a systematic, neutral and reproducible comparison of relevant statistical properties. Finally, we introduce a new open-source R package cases^a^ which provides convenient implementations for all investigated methods.

This work is structured as follows. The following section presents a motivational real-world data example. Different multiple comparison procedures and the design of the simulation study are then introduced in the subsequent section. Thereafter, the results of the simulation study and the analysis of the example data set are presented. A discussion of the results can be found in the final section.

## 2. Motivating example: Breast cancer diagnosis

For the motivating example, we utilize the ‘Breast Cancer Wisconsin (Diagnostic) Data Set’^b^ . In total, the dataset consists of 569 observations of digitized images of a fine needle aspirate of breast masses. Hereby, $n_{1}=212$ observations are breast cancer cases and $n_{0}=357$ are controls (fibrocystic breast masses). Thirty real-valued features were computed from the image data which describe characteristics of the cell nuclei present in the image. Several prior works have described the dataset and feature extraction methodology in detail.^14–17^ Two (independent) example scenarios are described below and analysed near the end of this work. For both scenarios, we assume that a high sensitivity is deemed more important compared to a high specificity because false negatives have more serious consequences than false positives.

### 2.1. Scenario A: Biomarker assessment

In this first example scenario, our goal is the assessment of the diagnostic accuracy of multiple biomarker candidates in a single study. In this example, three of the 30 available features have been selected before the study based on prior data and knowledge. The selected biomarker candidates are the most extreme area, compactness and concavity of any of the cell nuclei. To define a diagnostic test based on a continuous biomarker, a threshold needs to be specified in addition to allowing categorization into diseased and healthy subjects. However, the optimal threshold can usually only be specified with uncertainty before the study. In our example, we specify five threshold candidates for each biomarker, roughly corresponding to the 30%, 40%, 50%, 60% and 70% quantiles of each biomarker distribution. In this scenario, our framework will be used to investigate the diagnostic accuracy of the resulting 15 combinations of markers (3) and thresholds (5 per marker) simultaneously while adjusting for the resulting multiplicity.

### 2.2. Scenario B: Risk model evaluation

In modern medical applications, individual predictive features are commonly combined with risk prediction models. In this second example scenario, we evaluate several candidate models simultaneously. We split the dataset into 379 and 190 observations for model learning and evaluation, respectively, to emulate a prospective evaluation study after model development. In the development phase, five variants of the elastic net algorithm are employed, corresponding to five different values of the penalty mixing hyperparameter α∈{0,0.25,0.5,0.75,1} .^18,19^ For each of the five variants, the optimal penalty strength λ is found via 10-fold cross-validation. In the subsequent evaluation phase, the five optimized models ought to be assessed simultaneously. Because each model provides a predicted probability of cancer, the question arises of which probability threshold should be used in practice to make classifications. As a high sensitivity is targeted, the thresholds 10%, 20%, 30%, 40%, and 50% will be evaluated on the remaining ‘prospective’ evaluation data. In effect, in this scenario, our framework will be used to investigate the diagnostic accuracy of the resulting 25 combinations of risk models (5) and probability thresholds (5 per model) simultaneously while adjusting for the resulting multiplicity.

## 3. Methods

In the following, the statistical model and the hypotheses are presented. Subsequently, various approaches which allow for a multiplicity-adjusted analysis are then described. Finally, the design of the simulation study is outlined.

### 3.1. Statistical model and hypotheses

A sample of n individuals is assumed, divided into $n_{0}$ non-diseased and $n_{1}$ diseased (with $n=n_{0}+n_{1}$ ). We consider the within-subject design, that is, for each person i=1,…,n , the reference standard and m index tests are applied. The comparison of each test $T_{j}$ , j=1,…,m and reference standard D results in a true sensitivity ${\mathrm{se}}_{j}=P(T_{j}=1|D=1)$ and specificity ${\mathrm{sp}}_{j}=P(T_{j}=0|D=0)$ . Hereby, $T_{j}\in \{0,1\}$ indicates test-positive (1) and test-negative (0) and D∈{0,1} indicates diseased (1) and non-diseased (0) individuals, respectively. As stated in the introduction, the different index tests $T_{j}$ may be based on a single clinical parameter or biomarker, which is dichotomized based on different cut points. On the other hand, each $T_{j}$ may also be the result of a different complex prediction model based on high-dimensional input data (e.g. medical images). A mixture of simple and complex models is also possible.

We consider the two alternative hypotheses that true sensitivity and specificity are above a pre-specified minimum sensitivity and specificity, which are denoted by ${\mathrm{se}}_{0}$ and ${\mathrm{sp}}_{0}$ .^2^ This leads to the individual null hypotheses

(1) $$\begin{array}{r} H_{0,j}^{\mathrm{se}}:{\mathrm{se}}_{j}\leq {\mathrm{se}}_{0},\;H_{0,j}^{\mathrm{sp}}:{\mathrm{sp}}_{j}\leq {\mathrm{sp}}_{0} \end{array}$$

for each test $T_{j}$ . These two hypotheses are connected according to the intersection–union principle to the combined null hypothesis

(2) $$\begin{array}{r} H_{0,j}:\;H_{0,j}^{\mathrm{se}}\;\cup \;H_{0,j}^{\mathrm{sp}} \end{array}$$

per index test $T_{j}$ , j=1,…,m . The global null hypothesis for our multiple-testing problem then reads as

(3) $$\begin{array}{r} H_{0}:\;\overset{m}{\underset{j=1}{⋂}}H_{0,j}=\overset{m}{\underset{j=1}{⋂}}\left\{H_{0,j}^{\mathrm{se}}\;\cup \;H_{0,j}^{\mathrm{sp}}\right\} \end{array}$$

While we focus on one-sided hypotheses in this work, an extension to the analogue two-sided hypotheses is easily possible. Usual (maximum-likelihood) estimates of sensitivity ( ${\hat{se}}_{j}$ ) and specificity ( ${\hat{sp}}_{j}$ ) are calculated as

(4) $$\begin{array}{r} {\hat{se}}_{j}=\frac{n_{j,11}}{n_{1}}\;\text{and}\;{\hat{sp}}_{j}=\frac{n_{j,00}}{n_{0}} \end{array}$$

with $n_{j,11}$ as the number of diseased individuals with a positive test result and $n_{j,00}$ as the number of non-diseased individuals with a negative test result, each for test j . In the R package cases^*a*^, the estimates (4) are actually slightly shrunk towards 0.5 by default to prevent singular (co)variance estimates.^8^ The decision to retain or reject any null hypothesis can be made by means of a multiple comparison procedure as discussed in the next two sections.

### 3.2. Multiple comparison procedures

As a starting point for the data analysis, we define the usual Wald test statistics for the j th index test by

(5) $$\begin{array}{r} Z_{j}^{\mathrm{se}}=\frac{{\hat{\mathrm{se}}}_{j}-{\mathrm{se}}_{0}}{\sqrt{{\hat{\mathrm{se}}}_{j}(1-{\hat{\mathrm{se}}}_{j})/n_{1}}}\;\text{and}\;Z_{j}^{\mathrm{sp}}=\frac{{\hat{\mathrm{sp}}}_{j}-{\mathrm{sp}}_{0}}{\sqrt{{\hat{\mathrm{sp}}}_{j}(1-{\hat{\mathrm{sp}}}_{j})/n_{0}}} \end{array}$$

A level α test for the combined hypothesis $H_{j}$ (without adjustment for multiplicity) can easily be obtained. For this, both individual test statistics need to surpass an appropriate critical value $c_{\alpha }$ .^1,2^ In the simplest case, $c_{\alpha }=z_{1-\alpha }$ is the 1−α quantile of the standard normal distribution. With this choice, we can define the rejection criterion

(6) $$\begin{array}{r} \varphi_{j}=1\;⟺\;Z_{j}=\min(Z_{j}^{\mathrm{se}},Z_{j}^{\mathrm{sp}})>c_{\alpha }\;⟺\;Z_{j}^{\mathrm{se}}>c_{\alpha }\wedge Z_{j}^{\mathrm{sp}}>c_{\alpha } \end{array}$$

This approach gives approximate control of the type I error for $H_{0,j}$ under arbitrary parameter configurations $\left({\mathrm{se}}_{j},{\mathrm{sp}}_{j}\right)$ . Due to the asymptotic nature of the procedure, the type I error is often inflated for small sample sizes and proportions close to 0 or 1.^20^ Most importantly, even in the asymptotic case, it does not provide control of the family wise error rate (FWER) for $H_{0}$ which is defined as the probability to obtain at least one false positive rejection of one of the true null hypotheses $H_{0,j}$ , j∈1,…,m .

Various possibilities exist to allow for control of the FWER of which several are investigated in this work. All methods are based on existing approaches which, however, need to be adapted to the special structure of the hypothesis problem (3). The required adaptation is similar for all methods and implies a higher power compared to a native application. This is illustrated in detail based on corresponding comparison regions in the next section. In the following, we briefly describe all investigated methods.

- **No adjustment**: As outlined above, this naive approach is based on (6) and $c_{\alpha }=z_{1-\alpha }$ chosen to be the 1−α quantile of the standard normal distribution.
- **Bonferroni**: This well-known multiplicity correction can also be described by (6) but $c_{\alpha }=z_{1-\alpha /m}$ now corresponds to the standard normal quantile for an adjusted local significance level α/m .
- **maxT**: This asymptotic parametric approach is based on a multivariate normal approximation of the relevant vector of test statistics.^8^ The critical value $c_{\alpha }$ in (6) is calculated as an equicoordinate quantile of the multivariate normal distribution with expectation 0 and estimated correlation matrix $\hat{R}$ . To achieve the targeted error rates for the hypothesis problem (3) with this approach, $\hat{R}$ depends on the ‘estimated least-favourable parameter configuration (LFC)’, that is, the most likely LFC given the data. Taking into account the empirical correlation matrix $\hat{R}$ , this method is strictly more powerful compared to the Bonferroni approach if the decisions of the diagnostic tests are positively correlated.
- **Pairs bootstrap**: A nonparametric approach based on (6) whereby $c_{\alpha }$ is chosen to be the empirical 1−α quantile of a bootstrap sample of the maximum test statistic $Z=\max_{j}(Z_{j})=\max_{j}\min(Z_{j}^{\mathrm{se}},Z_{j}^{\mathrm{sp}})$ . For that matter, B bootstrap samples of the original data are drawn in a ‘paired’ fashion such that the original correlation structure of the problem is replicated. For our specific problem, each paired data re-draw i=1,…,B consists of one row $(D_{i},T_{i1},\ldots ,T_{im})\in \{0,1{\}}^{m+1},$ which is sufficient to calculate the required bootstrap parameter estimates and test statistics. Hereby, $D_{i}$ indicates the true disease status for the i th patient as assessed by the reference standard. For more information, we refer to a general introduction to this and the next bootstrap approach in the context of regression models.^21^
- **Wild bootstrap**: This approach is similar to the pairs bootstrap but the mechanism to draw new bootstrap samples is different.^21^ Here, we consider residuals of the binary indicator variables which indicate correct and wrong test decisions relative to the mean values per diagnostic test and subgroup, that is, relative to the empirical sensitivities or specificities. These residual values are resampled first and then multiplied by random weights (standard normal by default). As a result, the resampled data indicating a correct or wrong test decision are not binary and as such not on the scale of the original data. This approach is still investigated here as it has shown promising characteristics in a previous methodological study in the context of diagnostic accuracy studies.^12^
- **mBeta**: A Bayesian approach whereby two independent multivariate beta-binomial models are fitted for diseased and healthy subgroups.^22^ A rather particular loss function needs to be employed to achieve (frequentist) control of the FWER relevant to hypothesis problem (3). Firstly, a multivariate beta prior is assumed for the ( m -dimensional) vector of sensitivity parameters and the ( m -dimensional) vector of specificity parameters, respectively. These priors are specified weakly (little evidence) and conservatively (centred at 0.5) as m independent uniform (Beta(1,1)) distributions in all simulation settings. This prior leads to slight shrinkage of all 2m parameter estimates (posterior mean) towards 0.5 . This prior is then updated based on the observed multivariate binomial data.^22^ Lastly, an additional hyperparameter lfc_pr in the unit interval is employed which specifies the probability that an LFC occurs. To achieve frequentist type error control, the lfc_pr parameter is set to one by default. For an LFC, the posterior distribution is altered such that for each diagnostic test j=1,…,m , one of the parameters ${\mathrm{se}}_{j}$ , ${\mathrm{sp}}_{j}$ is assumed not to be covered by the respective credible interval. This affects the result of the subsequently employed numerical routine to derive a full, 2m -dimensional credible region based on the posterior draws. In contrast to all other techniques, the Bayesian mBeta approach does not provide p -values alongside test decisions.

All methods are implemented in the new R package cases. Implementation details can be checked in the open-source code and corresponding documentation. Besides the Bayesian approach, all methods are capable of providing adjusted p -values for decision making.

The above-mentioned hypotheses can be adapted to the case that all index tests $T_{j}$ are compared to a common comparator $T_{0}$ , that is, established diagnostic test with unknown diagnostic accuracy, instead of fixed performance thresholds ${\mathrm{se}}_{0}$ and ${\mathrm{sp}}_{0}$ . All statistical test procedures listed above are also capable of performing more general contrast tests and allow to specify non-inferiority or superiority margins. In the simulation study presented later, we, however, only consider the scenario described by equation (3).

### 3.3. Confidence and comparison regions

Besides multiplicity-adjusted test decisions and p -values, a (two-dimensional) uncertainty quantification is also important in diagnostic accuracy studies. Confidence regions are the multivariate counterpart of confidence intervals. In our case of m index tests, each with unknown sensitivity and specificity, we are interested in 2m -dimensional confidence regions $C=C_{1-\alpha }$ with coverage probability 1−α , that is, we require

(7) $$\begin{array}{r} \forall \theta =(\mathrm{se}\;\mathrm{se},\mathrm{sp}\;\mathrm{sp})=({\mathrm{se}}_{1},\ldots ,{\mathrm{se}}_{m},{\mathrm{sp}}_{1},\ldots ,{\mathrm{sp}}_{m})\in [0,1{]}^{2m}:\;P\left(\theta \in C\right)\geq 1-\alpha \end{array}$$

We focus our attention on rectangular confidence regions C which are the Cartesian product of test specific confidence regions $C_{j}=C_{j}^{\mathrm{se}}\times C_{j}^{\mathrm{sp}}$ with $C_{j}^{\mathrm{se}}\subset [0,1]$ and $C_{j}^{\mathrm{sp}}\subset [0,1]$ . For simplicity, we only consider one-sided intervals/regions in the following, with all upper limits equal to one.

The duality of confidence intervals and statistical tests for a single parameter is well-known. Similarly, we might expect that defining a statistical test by rejecting $H_{j}$ when both ${\mathrm{se}}_{0}\notin C_{j}^{\mathrm{se}}$ and ${\mathrm{sp}}_{0}\notin C_{j}^{\mathrm{sp}}$ from (2) gives us valid, multiplicity adjusted test decisions. While this approach indeed allows (approximate) control of the FWER, we will illustrate in the following that this procedure is conservative and can easily be improved.

The concept of comparison regions to display the uncertainty in sensitivity and specificity of a diagnostic procedure has been recently introduced.^23^ We define the region of interest $R=\{(\mathrm{se},\mathrm{sp})\in [0,1{]}^{2}:\mathrm{se}>{\mathrm{se}}_{0}\wedge \mathrm{sp}>{\mathrm{sp}}_{0}\}$ as the complement of $H_{0,j}$ in $[0,1{]}^{2}$ . A comparison (or decision) region for a single index test is a region $D_{j}$ such that defining a statistical test $\varphi_{j}$ via

(8) $$\begin{array}{r} \varphi_{j}=1\;⟺\;D_{j}\subset R \end{array}$$

allows control of the type I error rate. In other words, a statistical test decision based on the comparison region indicates a significant result if $D_{j}$ is completely contained in R . In contrast to prior work,^23^ we focus on rectangular regions of interest in this work and in addition introduce the concept of multiplicity-adjusted comparison regions.

The simplest way to illustrate the difference between confidence and comparison regions is the Bonferroni adjustment. For a single index test ( j=m=1 ), consider the region

(9) $$\begin{array}{r} A_{j,\alpha^{*}}=\left({\hat{\mathrm{se}}}_{j}-c_{\alpha^{*}}\sqrt{\frac{{\hat{\mathrm{se}}}_{j}(1-{\hat{\mathrm{se}}}_{j})}{n_{1}}},1\right]\times \left({\hat{\mathrm{sp}}}_{j}-c_{\alpha^{*}}\sqrt{\frac{{\hat{\mathrm{sp}}}_{j}(1-{\hat{\mathrm{sp}}}_{j})}{n_{0}}},1\right] \end{array}$$

with $c_{\alpha^{*}}=z_{1-\alpha^{*}}$ being the $1-\alpha^{*}$ standard normal quantile. Then, $C_{j,\alpha }=A_{j,\alpha /2}$ ( $\alpha^{*}=\alpha /2$ ) defines an (asymptotic) confidence region for $\theta_{j}=({\mathrm{se}}_{j},{\mathrm{sp}}_{j})$ . An adjustment for multiplicity is needed here, as both, sensitivity and specificity shall be covered by C with high probability, compare (7). On the other hand, the comparison region, as the statistical test for the co-primary endpoint problem, does not require an adjustment for α and hence $D_{j,\alpha }=A_{j,\alpha }$ ( $\alpha^{*}=\alpha$ ).

This directly extends to multiplicity-adjusted confidence and comparison regions when m>1 index tests are simultaneously evaluated. Here, the Bonferroni adjustment is $\alpha^{*}=\alpha /(2m)$ for the confidence region, as 2m parameters shall be covered. The adjustment for the corresponding comparison region, however, is only $\alpha^{*}=\alpha /m$ . All statistical methods considered in this work allow us to calculate multiplicity-adjusted confidence and comparison regions. For all procedures, one will find that $D_{\alpha }\subset C_{\alpha }$ and thus decisions based on confidence regions will not be dual to test decisions but rather more conservative.

**Figure 1.**
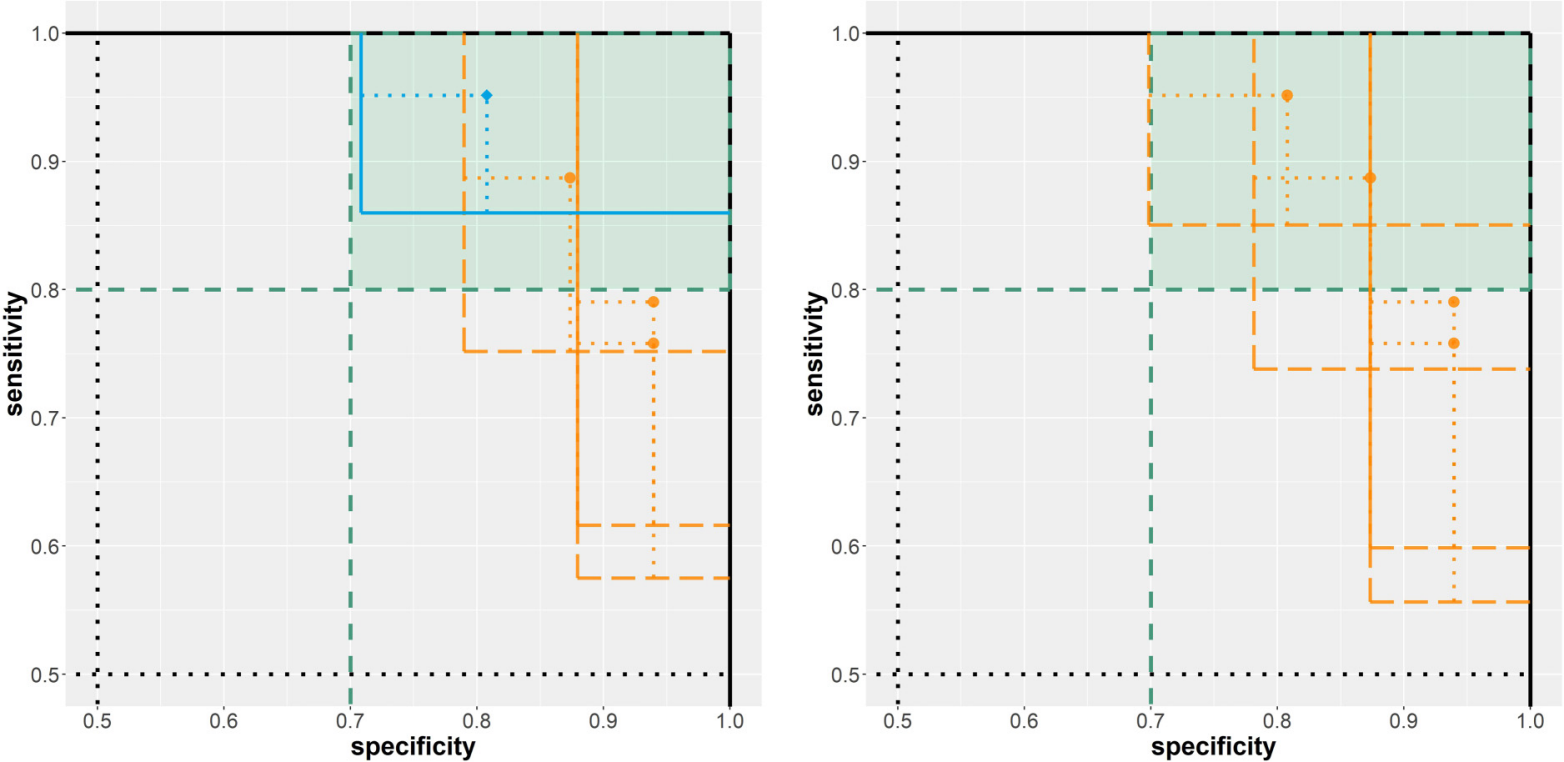
Exemplary analysis of synthetic example with four index tests. Region of interest ( ${\mathrm{se}}_{0}=0.8$ , ${\mathrm{sp}}_{0}=0.7$ ) is displayed in green. Left: comparison regions. Right: confidence regions. Solid/blue lines imply a rejected null hypothesis; dashed/orange lines imply a non-rejected null hypothesis.

Figure 1 illustrates this difference for a synthetic data set with $n_{1}=30$ cases and $n_{0}=90$ controls and m=4 index tests with varying accuracy. The region of interest R is displayed in the upper right-hand corner in green. In the left subfigure, multiplicity-adjusted comparison regions by the maxT-approach are indicated by solid lines around each point estimate $\left({\hat{\mathrm{se}}}_{j},{\hat{\mathrm{sp}}}_{j}\right)$ . The study conclusion in this case would be a rejection of the null $H_{0,j}$ only one of the four index tests ( j=1 , solid/blue lines) as $D_{1}\subset R$ but $D_{j}⊄R$ for all other tests j=2,3,4 (dashed/orange lines). The right subfigure reveals that a decision based on the corresponding (maxT-adjusted) confidence regions would have not resulted in any rejection of the null hypothesis as $C_{j}⊄R$ for all j=1,2,3,4 .

### 3.4. Simulation study

We perform a simulation study to evaluate and compare the multiple comparison procedures described in this work. Our primary goal is a comparison with regard to FWER and statistical power. The (disjunctive) statistical power is the probability of obtaining at least one rejection of a false null hypothesis $H_{0,j}$ . For that matter, two different data-generating mechanisms (‘LFC’ and ‘Biomarker’) are investigated and outlined in the following. The key features of the simulation study are described in Table 1 following the aims, data-generating mechanisms, estimands, methods, and performance measures (ADEMP) framework.^24,25^

**Table 1.** Key features of simulation study.

Feature	Description
Aims	Comparison of different MCPs for diagnostic accuracy studies with co-primary endpoints
Data generating mechanisms	Different scenarios (w.r.t. sample size, control-to-case ratio, number of tests, parameter values, correlation structure) in two distinct settings (‘LFC’ and ‘Biomarker’)
Methods of analysis	Multiple comparison procedures for hypothesis problem (3)
Performance measures	FWER and (disjunctive) power
Number of repetitions	$n_{\mathrm{sim}}=10,000$ per scenario

MCP: multiple comparison procedure; FWER: family-wise error rate.

For both simulation settings, many different scenarios are considered which are described by a number of design parameters (setting, sample size, control-to-case ratio, number of tests, parameter values, and correlation structure). For each scenario, $n_{\mathrm{sim}}=10,000$ synthetic datasets were generated. The simulation study is conducted such that all methods are applied to the exact same synthetic datasets. The standard (Monte-Carlo) error for estimated proportions p such as the FWER or statistical power is $(p(1-p)/n_{sim}{)}^{1/2}$ , which is bounded by 0.005 for $n_{\mathrm{sim}}=10,000$ . In all simulation scenarios, we investigated sampling with fixed sample sizes $n_{0}$ , $n_{1}$ , which corresponds to a simple case-control within-subjects study design.

All numerical experiments presented in this work are reproducible by means of the new R package cases^*a*^. Instruction for that matter is provided via a GitHub repository^c^ which also contains a separate simulation report with more detailed results and additional sensitivity analyses^d^ .

As the source code for all statistical methods is available in the cases package, this also serves as a documentation of implementation-related details. To reduce the computational demand of the simulation study, we consider only the comparison of m candidate tests to fixed performance criteria ${\mathrm{se}}_{0}={\mathrm{sp}}_{0}$ . The cases package furthermore supports a comparison of m candidate tests to the unknown sensitivity and specificity of a comparator and other relevant contrast tests. The simulations were conducted in R (version 4.2.1) with the help of the batchtools^e^ package.

#### 3.4.1. LFC setting

In the first setting, a single synthetic data set consists of two separate samples for diseased and healthy. For that matter, two different multivariate Binomial models are employed to generate binary data matrices $Q^{1}$ and $Q^{0}$ . These matrices have entries

(10) $$Q_{ij}^{g}=1(T_{ij}=D_{i}),\;i=1,\ldots ,n,\;j=1,\ldots ,m,\;g=0,1$$

whereby $Q_{ij}^{g}$ indicates if the j th diagnostic test (data-based decision rule) $T_{j}$ under consideration correctly predicted the i th disease status $D_{i}$ in subgroup g∈{0,1} . The multivariate binomial distribution in general has $2^{m}$ parameters but reduced representations do exist which can be specified by the mean vector and correlation matrix. We utilize the R package bindata^f^ to generate the data $Q^{\mathrm{se}}$ , $Q^{\mathrm{sp}}$ with given first moments sese , spsp , respectively.^26^

In this scenario, we focus on LFCs which are defined as parameters which maximize the FWER. As discussed in prior work,^6^ an LFC in this case is defined by mean vectors sese , spsp with entries

(11) $$\begin{array}{r} {\mathrm{se}}_{j}=\left\{ \begin{array}{ll} {\mathrm{se}}_{0}, & b_{j}=1\\ 1, & b_{j}=0 \end{array}\right.,\;{\mathrm{sp}}_{j}=\left\{ \begin{array}{ll} {\mathrm{sp}}_{0}, & b_{j}=0\\ 1, & b_{j}=1 \end{array}\right. \end{array}$$

whereby ${\mathrm{se}}_{0}$ , ${\mathrm{sp}}_{0}$ are the parameter thresholds which define the hypotheses (1) and $b=(b_{1},\ldots ,b_{m})\in \{0,1{\}}^{m}$ is a fixed binary vector. For each index j , we thus have either ${\mathrm{se}}_{j}$ or ${\mathrm{sp}}_{j}$ exactly at the boundary of the null hypothesis and the other parameter equal to one. For each of the two endpoints, a correlation matrix is specified in addition. To limit computational resources, we restricted the attention to equicorrelation between non-degenerated parameters, that is, $\rho_{jj^{\prime }}^{se}=\rho^{\mathrm{se}}$ for all indices $j\neq j^{\prime }$ with ${\mathrm{se}}_{j}\neq 1$ and ${\mathrm{se}}_{j^{\prime }}\neq 1$ .

#### 3.4.2. Biomarker setting

The LFC setting discussed in the last section allows us to assess worst-case error rates. However, LFCs are rarely representative of real-world situations.^8^ The goal of this second simulation study is thus to evaluate the multiple comparison procedures under more realistic parameter configurations.

To generate a single synthetic data set, we start by simulating l continuous biomarkers from a multivariate binormal ROC model.^27^ This means, that we consider multivariate diagnostic markers $V=(V_{1},\ldots ,V_{l})$ such that

(12) $$\begin{array}{r} V\;|\;D=1∼N(\mu^{1},R^{1}),\;V\;|\;D=0∼N(0,R^{0}) \end{array}$$

Hereby, $\mu_{k}^{1}$ , is the mean of marker k in the diseased ( D=1 ) population. For simplicity, we assume a mean $\mu_{k}^{0}=0$ for all k=1,…,l in the healthy population ( D=0 ) and a variance of one in both populations. If we aim for a certain AUC of marker k , this can be achieved by solving

(13) $$\begin{array}{r} {\mathrm{AUC}}_{k}=\Phi \left(\frac{\mu_{k}^{1}}{\sqrt{2}}\right) \end{array}$$

for $\mu_{k}^{1}$ which has the solution $\mu_{k}^{1*}=\sqrt{2}\Phi^{-1}({\mathrm{AUC}}_{k})$ .^27^ Hereby, Φ is the cumulative distribution function of the standard normal distribution.

These continuous scores $V_{k}$ are split at different cut points $c_{j}$ to obtain an overall list of diagnostic tests $T_{j}=1(V_{k_{j}}>c_{j})$ , j=1,…,m with m≥l . The resulting decision rules have true parameters

(14) $$\begin{array}{r} {\mathrm{se}}_{j}=\Phi \left(\mu_{k_{j}}^{1}-c_{j}\right),\;{\mathrm{sp}}_{j}=1-\Phi \left(-c_{j}\right) \end{array}$$

depending on the cut point $c_{j}$ . Alternatively, this process can also be identified as thresholding risk model scores to obtain different prediction models. The correlation matrices $R^{0},R^{1}$ in (12) have a simple structure (e.g. equicorrelation) in our simulation study. Together with the chosen cut points, this induces a certain correlation structure for the actual binary decision rules. Naturally, two (close) nearby cut points, induce (highly) similar predictions and thus a (highly) positive correlation between estimates.

## 4. Results

### 4.1. Simulation study

#### 4.1.1. LFC setting

**Figure 2.**
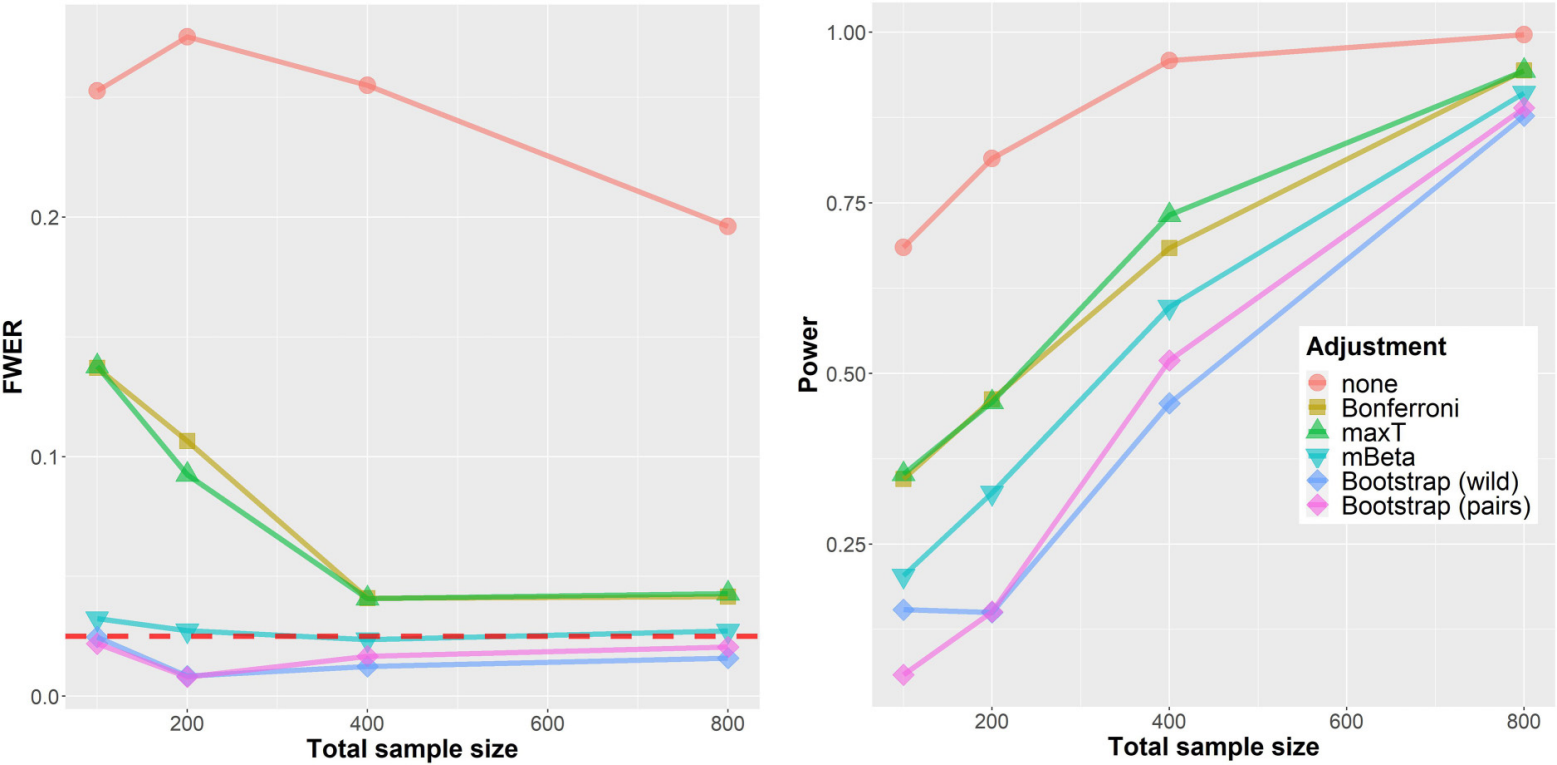
Simulation results for the ‘LFC’ setting. Left: FWER. Right: Power. The horizontal axis shows the total sample size (cases and controls $n=n_{1}+n_{0}$ ) in both subplots. LFC: least-favourable parameter configuration; FWER: family-wise error rate.

The simulation results from the LFC setting are displayed in Figure 2. On the left, the FWER is shown depending on the total sample size $n=n_{1}+n_{0}$ for a control-to-case ratio of 3:1 and m=10 index tests. The parameter values are set to an LFC with ${\mathrm{se}}_{0}={\mathrm{sp}}_{0}=0.8$ , compare (11). The red dashed vertical line shows the (one-sided) target significance level of α=0.025 .

First, applying no multiplicity adjustment at all clearly leads to a vastly increased FWER of 20% and more compared to the target significance level of α=0.025 . Second, the asymptotic methods (maxT, Bonferroni) are capable of controlling the FWER for large n but fail to do so for small n . For n=100 , both methods show an increased FWER of up to 10%, declining to around 5% for n≥800 . Third, the bootstrap approaches (pairs and wild) and the Bayesian mBeta approach all control the FWER for all but the lowest sample size of n=100 .

The right part of Figure 2 shows the (disjunctive) power for the same data instances after dropping the minimal acceptance criteria ${\mathrm{se}}_{0},{\mathrm{sp}}_{0}$ both to 0.75 . The ordering of methods stays the same as for the FWER analysis, as expected. The gap between mBeta and Bootstrap approaches is somewhat more pronounced than before.

Further analyses (simulation report^*d*^, section 2.1) indicate, that these findings for FWER and power remain qualitatively similar when only m=5 index tests are considered or when the correlation structure between diagnostic test results is changed. When the control-to-cases ratio is changed to 10:1 , all curves are essentially shifted. To obtain a similar power as before, a higher total sample size is required due to the dependence on $\min(n_{1},n_{0})$ . Changing ${\mathrm{se}}_{0}$ from 0.8 to 0.9 results in an increased FWER for small sample sizes but the qualitative observations regarding the asymptotic behaviour still persist. For instance, when changing ${\mathrm{se}}_{0}$ to 0.9 (from 0.8) in the scenario shown in Figure 2, then the FWER for the parametric approaches (maxT and Bonferroni) is increased to 0.22 (from 0.14) for n=100 . Further details are provided in the supplementary simulation report^*d*^ (section 2.1.2). In general, the small sample FWER of the non-parametric (pairs bootstrap and wild bootstrap) and Bayesian (mBeta) approaches are much closer to the nominal level compared to the parametric methods.

In an additional experiment, the influence of different values of the prior parameter lfc_pr (0, 0.5, 1) of the mBeta approach was explored (simulation report^*d*^, section 2.3.2). Here, we observed that deviations from the default value one led to vastly increased error rates, as expected.

#### 4.1.2. Biomarker setting

**Figure 3.**
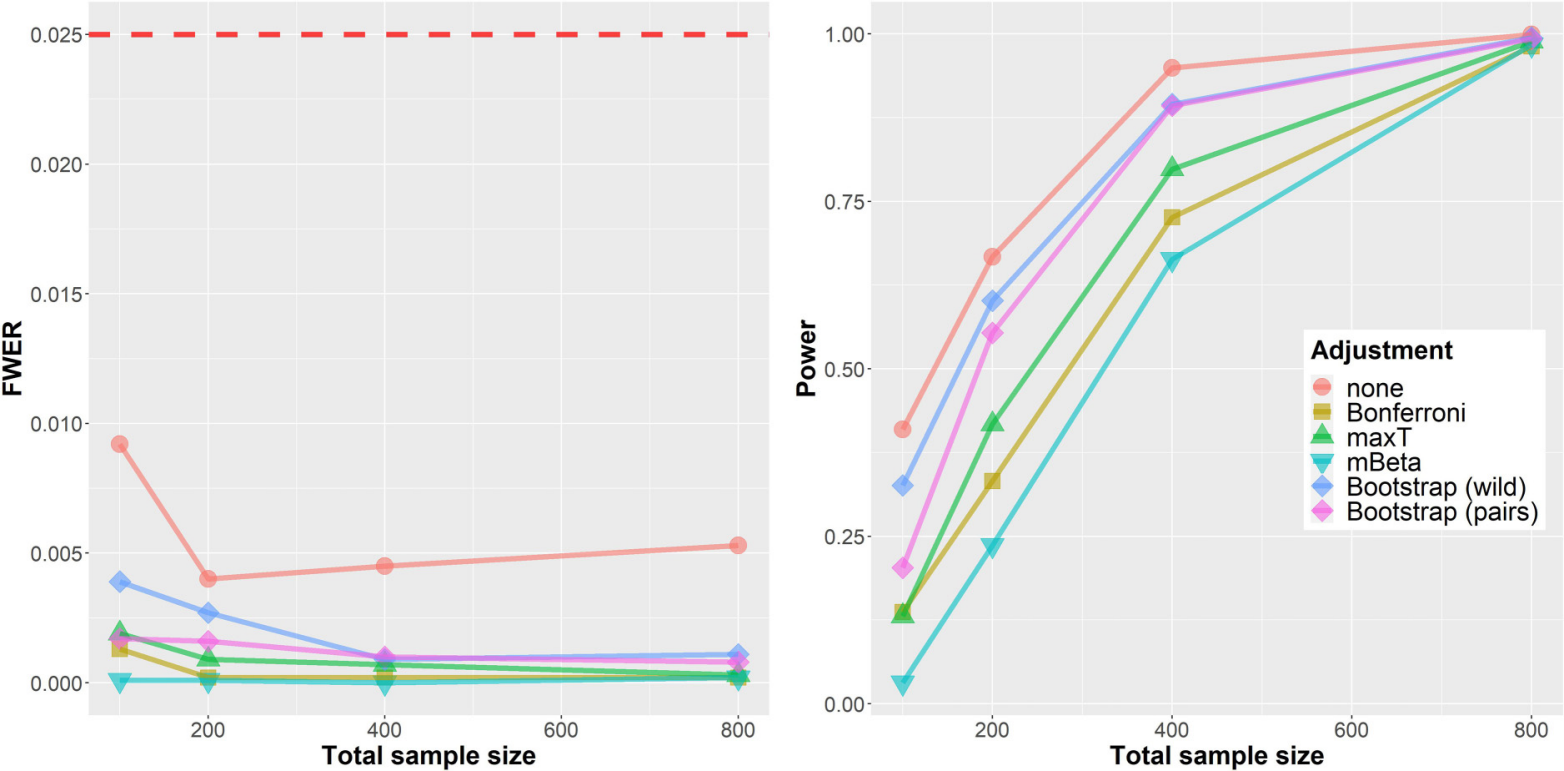
Simulation results for the ‘Biomarker’ setting. Left: family-wise error rate (FWER). Right: Power. The horizontal axis shows the total sample size (cases and controls $n=n_{1}+n_{0}$ ) in both subplots.

Figure 3 shows results from the simulation in the more realistic biomarker setting. In contrast to the worst-case assessment in the last section, the FWER is below 0.005 for all methods and nearly all sample sizes and as such far below the target significance level of α=0.025 . This behaviour is expected based on previous work.^6^

Regarding statistical power the ordering of methods is mostly similar to the situation in the LFC setting. One important difference is the much better performance of both Bootstrap approaches which seem to adapt much better to the underlying generative distribution in this situation. Further results are shown in the separate simulation report (section 2.2).^*d*^

In a sensitivity analysis, the deviation from the bi-normal ROC model was investigated. For this matter, diagnostic markers were generated in a similar fashion but from a bi-exponential ROC model. This resulted in increased rejection rates for all procedures, most noticeably for the parametric approaches (no adjustment, Bonferroni and maxT). However, the FWER was still far below the nominal significance level in all simulation scenarios. Details are provided in the simulation report (section 2.3.1).^*d*^

### 4.2. Analysis of motivating example

To conclude, we apply our methodology to real-world data and interpret the results. For that matter, the pairs bootstrap approach is applied in the two motivating example scenarios introduced at the beginning of this work. To formalize our requirement that high sensitivity is prioritized, we define the minimal acceptance criteria as ${\mathrm{se}}_{0}=0.9$ and ${\mathrm{sp}}_{0}=0.7$ . The entire analysis can be reproduced by following the corresponding vignette of the new R package cases.

#### 4.2.1. Scenario A: Biomarker assessment

**Figure 4.**
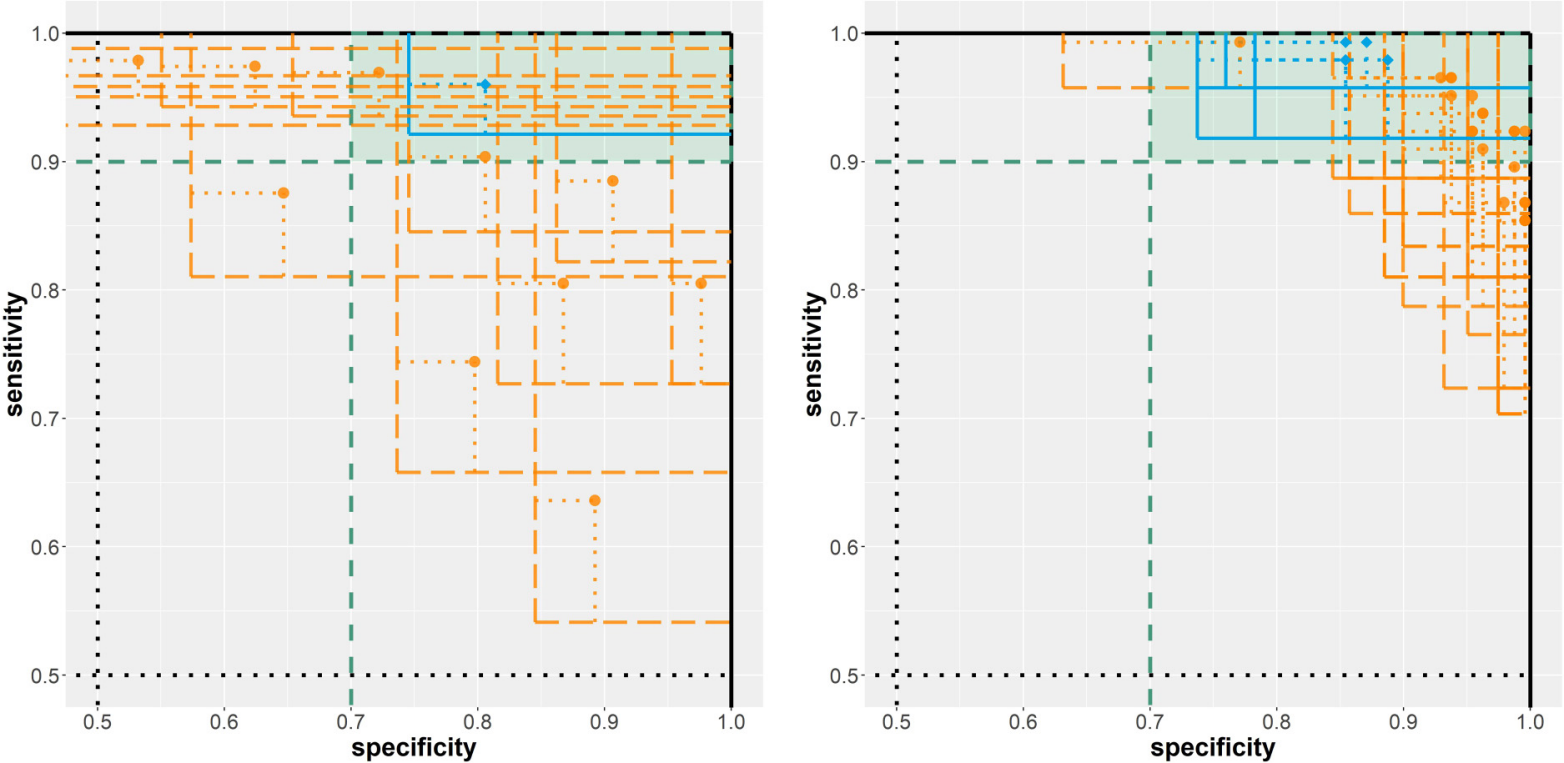
Results from the analysis of the two breast cancer diagnosis example scenarios. Left: biomarker assessment (scenario A). Right: risk model evaluation (scenario B). Solid/blue lines imply a rejected null hypothesis; dashed/orange lines imply a non-rejected null hypothesis.

The most promising of the 15 investigated classification rules in terms of diagnostic accuracy on the evaluation data (212 cases and 357 controls) is the maximal area of any present cell nucleus with a threshold of $700\mu m^{2}$ applied for categorization into cases ( $>700\mu m^{2}$ ) and controls ( $\leq 700\mu m^{2}$ ). This rule achieves an empirical sensitivity of 96.0% and an empirical specificity 80.6% . The multiplicity-adjusted lower comparison bounds are 92.1% and 74.6% , respectively. As both lower bounds are larger than their respective acceptance criteria, the co-primary null hypothesis can be rejected for this test candidate. This is visualized in Figure 4 (left). None of the other comparison regions is completely contained in the region of interest corresponding to the alternative hypothesis. In effect, the null hypothesis cannot be rejected for any other test candidate even though individual sensitivity and specificity estimates of other candidates are larger than those reported above.

#### 4.2.2. Scenario B: Risk model evaluation

In total, 25 risk prediction models were assessed on the evaluation data (71 cases, 119 controls). For four models, the null hypothesis se≤0.9∨sp≤0.7 can be rejected as these models have lower comparison bounds for sensitivity and specificity that are greater than the corresponding acceptance criteria. Empirical sensitivities of these models lie between 99.3% and 97.9% and specificities between 88.8% and 85.4% . This is visualized in Figure 4(right). These four classifiers all result from thresholding different model risk scores at the lowest probability threshold of 0.10 . Because all four models are deemed acceptable regarding their discriminatory performance, other criteria may influence the final model decision. For instance, the most parsimonious model can be chosen to increase interpretability. In this example, the lasso fit (elastic net mixing parameter α=1 ) results in only 11 non-zero regression coefficients and is much sparser than the three competitors (with α<1 ) which have 18, 19 and 23 non-zero coefficients, respectively.

## 5. Discussion

In this work, we investigated statistical inference methods for diagnostic accuracy studies with multiple index tests and co-primary endpoints sensitivity and specificity. Multiplicity corrections are relevant in this context as omitting a suitable correction can otherwise induce overoptimistic results and inflated error rates. While a control is easily possible using a traditional (FWER) correction for all 2m parameters ( m index tests with unknown sensitivity and specificity), this approach is too conservative for the co-primary endpoint analysis. Several multiple comparison procedures can be adapted to the specific testing problem such that this conservative nature is resolved. This difference was illustrated by explicitly contrasting confidence regions and comparison regions. Only the latter allows for a two-dimensional uncertainty quantification which is dual to the statistical test result.

We conducted an extensive simulation study to compare five multiple comparison procedures with regards to FWER and statistical power. All five procedures are capable of controlling the FWER at least asymptotically. The Bonferroni adjustment and maxT approaches both need quite large sample sizes to reach the target significance level. This, however, depends crucially on the control-to-cases ratio. A more skewed class distribution results in higher sample size demand. The Bayesian mBeta approach and the two Bootstrap (pairs and wild) procedures managed to control the FWER much better under LFCs. Based on the simulation study, we recommend the use of the pairs bootstrap approach. This approach yielded good FWER control for small sample sizes and competitive power in realistic scenarios. The wild bootstrap approach has a very similar performance, but the method is more complex, has more design choices, and was not originally designed to be used for binary data. For larger sample sizes, the maxT approach is a competitive alternative and easier to apply compared to the bootstrap procedures. The Bonferroni method is by far the easiest method to apply but too conservative in some situations and as such suboptimal.

In this work, we focused on the FWER and the statistical power. In our framework, point estimates are slightly shrunk due to the implemented minor regularization to avoid singular (co)variance estimates. However, point estimates were not adjusted for multiplicity. It has been demonstrated that the maxT approach can be utilized to obtain median-conservative point estimators.^8,6^ This generic approach could also be adapted to the other multiple comparison procedures.

All investigated methods can be adapted to the case that not only two (diseased and healthy) subpopulations need to be distinguished but more than two subpopulations, for example, different disease severities. This capability is already implemented in the cases package. We expect that the performance will then crucially depend on the prevalence of the smallest subpopulation. This should, however, be confirmed in dedicated simulation studies.

## Notes

a https://CRAN.R-project.org/package=cases (last accessed 1 December 2023).

b https://archive.ics.uci.edu/ml/datasets/breast+cancer+wisconsin+(diagnostic) (last accessed 1 December 2023)

c https://github.com/maxwestphal/cases_simstudy (last accessed 1 December 2023).

d https://maxwestphal.github.io/cases_simstudy/ (last accessed 1 December 2023).

e https://CRAN.R-project.org/package=batchtools (last accessed 1 December 2023).

f https://CRAN.R-project.org/package=bindata (last accessed 1 December 2023).
